# Effects of copper occupancy on the conformational landscape of peptidylglycine α-hydroxylating monooxygenase

**DOI:** 10.1038/s42003-018-0082-y

**Published:** 2018-06-25

**Authors:** Sweta Maheshwari, Chizu Shimokawa, Katarzyna Rudzka, Chelsey D. Kline, Betty A. Eipper, Richard E. Mains, Sandra B. Gabelli, Ninian Blackburn, L. Mario Amzel

**Affiliations:** 1 Department of Biophysics and Biophysical Chemistry, Johns Hopkins University School of Medicine, Baltimore, MD 21205 USA; 2 Department of Chemistry, Kurume University School of Medicine, Kurume, Fukuoka 830-0011 Japan; 3 Division of Environmental and Biomolecular Systems, School of Medicine, Oregon Health and Sciences University, Portland, OR 97239 USA; 4 Department of Neuroscience, University of Connecticut, Farmington, CT 06030 USA; 5 Department of Medicine, The Johns Hopkins University School of Medicine, Baltimore, MD 21205 USA; 6 Department of Oncology, The Johns Hopkins University School of Medicine, Baltimore, MD 21287 USA

## Abstract

The structures of metalloproteins that use redox-active metals for catalysis are usually exquisitely folded in a way that they are prearranged to accept their metal cofactors. Peptidylglycine α-hydroxylating monooxygenase (PHM) is a dicopper enzyme that catalyzes hydroxylation of the α-carbon of glycine-extended peptides for the formation of des-glycine amidated peptides. Here, we present the structures of apo-PHM and of mutants of one of the copper sites (H107A, H108A, and H172A) determined in the presence and absence of citrate. Together, these structures show that the absence of one copper changes the conformational landscape of PHM. In one of these structures, a large interdomain rearrangement brings residues from both copper sites to coordinate a single copper (closed conformation) indicating that full copper occupancy is necessary for locking the catalytically competent conformation (open). These data suggest that in addition to their required participation in catalysis, the redox-active metals play an important structural role.

## Abstract (web summary)

Sweta Maheshwari et al. present X-ray crystal structures of the two-copper enzyme peptidylglycine α-hydroxylating monooxygenase and three inactive mutant forms. They show that full copper occupancy is needed to maintain the catalytically competent (open) conformation of the enzyme.

## Introduction

Secreted peptides function as hormones, neurotransmitters, and growth factors. In the animal kingdom, many of these peptides—most of them produced by enzymatic cleavage of large precursors—must be amidated at their carboxy terminus to exhibit full biological activity. Surprisingly, these amides are not generated by an amination reaction. Instead, precursors or intermediates with a glycine at their C-terminus are transformed into active, terminally amidated des-glycine hormones by oxidative cleavage of the glycine N-Cα bond^1–5^. Two enzymes, peptidylglycine α-hydroxylating monooxygenase (PHM; EC 1.14.17.3) and peptidyl-α-hydroxyglycine α-amidating lyase (PAL; EC 4.3.2.5) working sequentially are the only proteins known to catalyze this reaction. A gene encoding active, bifunctional, integral membrane peptidylglycine α-amidating monooxygenase (PAM) was recently identified in *Chlamydomonas reinhardtii*, a unicellular green alga, raising the possibility that PAM existed in the last eukaryotic common ancestor^6,7^. In *C. reinhardtii*, as in placozoans and sponges—organisms that lack both nervous and endocrine systems— and in most animals, the PHM catalytic core follows an N-terminal signal sequence and is followed by the PAL catalytic core, a single transmembrane domain and an unstructured cytosolic domain^4,5,7–10^. The PAM gene appears to have duplicated and in some organisms, including *Drosophila melanogaster*, PHM and multiple PAL proteins are encoded by separate genes^7^. It is now clear that a PAM protein that requires ascorbate, copper, and molecular oxygen appeared before the evolution of the nervous system.

PHM, a two Cu enzyme, catalyzes the stereospecific hydroxylation of the glycine Cα of peptidylglycine substrates. PAL, a Zn-containing enzyme, completes the reaction, yielding the α-amidated peptide product plus glyoxylate. Both domains have broad substrate specificity: peptides with all 20 amino acid amides have been isolated^3^. The cloning and successful expression of PHM, PAL, and bifunctional PAM have made detailed structural and functional studies possible^11–15^. The structures of the PHM and PAL domains of rat PAM, alone and in complexes with substrates, inhibitors, and other ligands, have been determined^11–15^. Other techniques used to study PHM have ranged from kinetics and kinetic isotope effect measurements^16–20^, to inhibitor design^21–24^, spectroscopy^25–29^ including X-ray absorption spectroscopy (XAS)^17,30–33^, and computational studies^11,34–36^. These studies have provided significant insights into the mechanism of both domains, especially of PHM.

The reaction carried out by PHM—hydroxylation of an aliphatic carbon—is chemically highly sophisticated. The two copper atoms in PHM, Cu_H_, and Cu_M_ (located in the N- and C-subdomains, respectively), each use a single reducing equivalent from ascorbate to catalyze the reduction of molecular oxygen for the hydroxylation of the Cα of glycine at the carboxy-terminus of the peptide substrate^37,38^. PHM is one of a limited number of enzymes that require copper for catalytic function; the residues that bind Cu_H_ and Cu_M_ are perfectly conserved from *C. reinhardtii* to human PHM. The transporters and chaperones needed to utilize copper in the secretory pathway are also highly conserved, suggesting an equally sophisticated metalation mechanism. The catalytic chemistry must therefore proceed within the confines of a complex cellular transport machinery, with the overall functioning of the system needed to balance the requirements of selective metalation with the structural determinants of catalytic function.

The studies enumerated above, especially X-ray diffraction, revealed numerous ancillary properties of PHM that may or may not be necessary for catalysis: Cu_H_ and Cu_M_ are 11 Å apart in both the reduced and the oxidized states; the peptide substrate provides the path for the electron transfer; Cu_H_ has an empty coordination site that remains empty even in the presence of high concentrations of small-molecule copper ligands; O_2_ binds only to Cu_M_; O_2_ binds with end-on geometry and only in the presence of substrate; H_2_O_2_ can bypass the requirement for O_2_ and for an additional source of reducing equivalents but binds Cu_M_ with side-on geometry. It is unknown whether these highly specific features are absolute mechanistic requirements or instead only evolutionary events that accompanied organismic specialization. A mechanism as sophisticated as that of PHM provides a unique platform for uncovering the differences among the evolution of the chemical requirements for product production, the constraints on metal ion coordination imposed by the metalation machinery, and those resulting from subsequent evolutionary events. The highly conserved Cu_H_ and Cu_M_ sites in PHM suggest that many of these features may be essential for function.

Information necessary to address these questions includes the effect of missing copper in one or both sites and the effect of modifying the coordination of Cu_H_ on the structure, activity and other properties of PHM. Cu_H_ is coordinated by three highly conserved histidine residues: H107, H108, and H172. Mutation of any one of these residues to alanine results in an inactive enzyme^1,39,40^. This loss of activity may be due to different consequences of the mutations. For example, do the mutations weaken or prevent Cu binding to the modified Cu_H_ site, with loss of one of the two required electrons for O_2_ reduction? Or, do the mutant proteins, even though they have an intact Cu_M_ site—the site of substrate binding—fail to bind substrate? In this work, we describe structural studies on rat PHM aimed at addressing some of these important questions. Crystallographic data on apo wild-type PHM and on several crystal forms of three Cu_H_ mutants (H107A, H108A, and H172A) presented here show that the copper ions are required not only for the catalytic steps but also for substrate binding and for locking the overall conformation of PHM in a configuration that is catalytically active. Absence of copper, or modified copper coordination, has a major effect on the overall flexibility of PHM, allowing the molecule to adopt conformations that have not been observed with wild-type PHM.

## Results

### Structure of rat apo-PHM

**Fig. 1:**
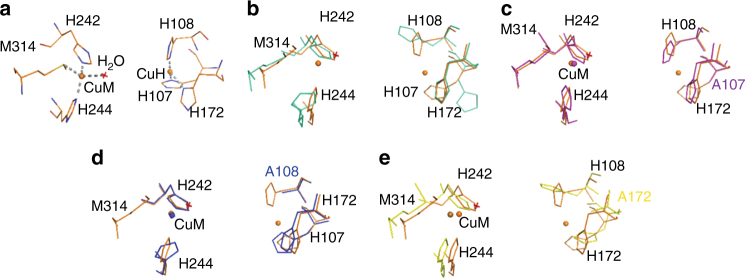
Geometry of the Cu_H_ and Cu_M_ sites in the structures of apo-PHM and PHM mutants-H107A, H108A and H172A. **a** Cu coordination in wild-type PHM (PDB ID: 1PHM), shown in orange. Cu_H_ and Cu_M_ of wild-type PHM are shown in orange spheres. Water molecule is shown as red crossmark. **b** Alignment of Cu coordinating residues of wild-type PHM with those of apo-PHM (cyan). Cu_H_ and Cu_M_ are absent in apo-PHM. **c** Alignment of wild-type PHM with H107A-PHM (magenta). Cu_H_ is absent in H107A-PHM and CuM is shown as magenta sphere. **d** Alignment of wild-type PHM with H108A-PHM (blue). Cu_H_ is absent in H108A-PHM and CuM is shown as blue sphere. **e** Alignment of wild-type PHM with H172A-PHM (yellow). Cu_H_ is absent in H172A-PHM and CuM is shown as yellow sphere

All of the PHM structures published to date contain two copper ions. To assess the possible structural role of copper, the structure of apo-PHM (PDB ID: 5WKW), which lacks bound copper, was determined to a resolution of 1.8 Å (Table 1). The final *R*-values are *R*_work_ = 20.9% and *R*_free_ = 27.3%. As expected, there was no density for either of the two coppers—Cu_H_ or Cu_M_ (Fig. 1). Other than this, the structure is strikingly similar to the wild-type holoenzyme (PDB ID: 1PHM) (RMSD 1.6 Å for 1214 main chain atoms), with the largest differences restricted to the loops connecting the β-strands (Supplementary Figure 1A).

**Table 1:** Data collection and refinement statistics

	Apo-PHM PDB ID: 5WKW	H107A-PHM PDB ID: 6ALV	H108A-PHM PDB ID: 6AO6	H172A-PHM PDB ID: 6AMP	H107A-PHM+citrate PDB ID: 5WJA
*Data collection*					
Space group	P2_1_2_1_2_1_	P2_1_2_1_2_1_	P2_1_2_1_2_1_	P2_1_2_1_2_1_	P2_1_
Cell dimensions					
* a*, *b*, *c* (Å)	58.72, 66.56, 70.18	69.04, 68.85, 81.53	69.12, 69.84, 81.99	59.42, 66.36, 69.79	59.54, 100.61, 101.58
* α*, *β γ* (°)	90, 90, 90	90, 90, 90	90, 90, 90	90, 90, 90	90.00, 89.98, 90.00
Resolution (Å)	50.00–1.79 (1.82–1.79)	50.0–3.50 (3.56–3.50)	52.85–3.30 (3.38–3.30)	50.0–2.48 (2.57–2.48)	30–2.3 (2.34–2.30)
*R*_sym_	0.078 (0.387)	0.12 (0.70)	0.062 (0.671)	0.068 (0.037)	0.082 (0.853)
*I* / σ*I*	38.39 (2.58)	13.43 (2.19)	10.1 (1.10)	25.32 (2.43)	21.34 (2.00)
CC(1/2)	NA	NA	0.99 (0.42)	NA	0.99 (0.82)
Completeness (%)	99.1 (92.0)	92.2 (88.9)	99.3 (45.5)	99.1(95.5)	100 (100)
Redundancy	7.4 (4.2)	3.6 (2.7)	2.95 (2.51)	4.5 (3.7)	4.2 (4.2)
No. unique reflections	26454	4848	8535	10124	52994
No. total reflections	195741	17348	25247	45581	223549
Source	FR-E Superbright/Saturn944	FR-E Superbright/Saturn944	APS Beamline 23-ID-B	FR-E Superbright/Saturn944	APS Beamline 23-ID-B
Wavelength (Å)	1.54	1.54	1.033	1.54	1.033
*Refinement*					
Resolution (Å)	48.30–1.79 (1.83–1.78)	52.69–3.50 (3.59–3.50)	53.17–3.30 (3.38–3.30)	48.09–2.48 (2.54–2.48)	71.48–2.30 (2.36–2.30)
No. reflections	24960 (1691)	4343 (187)	7507 (267)	9583 (650)	26271 (1815)
*R*_work/_ *R*_free_	0.20/0.27 (0.29/0.39)	0.27/0.29 (0.52/0.61)	0.18/0.24 (0.25/0.34)	0.21/0.29 (0.32/0.40)	0.20/0.27 (0.29/0.39)
No. atoms					
Protein	2489	2402	2428	2394	4916
Water	245	—	22	12	140
B-factors					
Protein	29.02	47.62	85.81	50.7	53.15
Ligand/ion	45.85	45.35	80.2	90.4	58.90
Water	36.20	—	61.82	39.8	49.70
R.m.s deviations					
Bond lengths (Å)	0.015	0.013	0.010	0.013	0.012
Bond angles (°)	1.81	1.73	1.46	1.69	1.78

### Structures of the PHM mutants (H107A, H108A, and H172A)

The structures of each of the Cu_H_ site PHM mutants were determined by X-ray diffraction in the absence and in the presence of 1–3 mM citrate, used as an additive to improve resolution. None of the crystallization conditions included Cu^2+^. Only the structures of crystals that diffracted to the highest resolution were fully refined and are reported here. All structures were determined without the addition of ascorbate and it is therefore expected that all observed coppers are in the oxidized state (Cu^2+^).

### Structure of the H107A-PHM mutant

The crystals of the Cu_H_ mutant, H107A-PHM (PDB ID: 6ALV), grown in the conditions used for wild-type PHM, diffract to only 3.5 Å resolution. Nevertheless, the structure was determined by molecular replacement and refined to an *R*-value of 27.6% (*R*-free = 29.7%) with excellent geometry (Table 1). The structure, except for the lack of Cu^2+^ in the Cu_H_ site, is very similar to that of wild-type PHM (RMSD = 0.42 Å for 283 Cα carbons) (Fig. 1c). Despite lacking its Cu^2+^, the rest of the Cu_H_ site shows little change except for the absence of the side chain of residue 107. The mutant does have Cu^2+^ in the Cu_M_ site and the geometry of the coordination is almost identical to that of wild-type PHM (Fig. 1c). The main difference is a small (<0.5 Å) narrowing of the space between the two domains (Supplementary Fig. 1B).

**Fig. 2:**
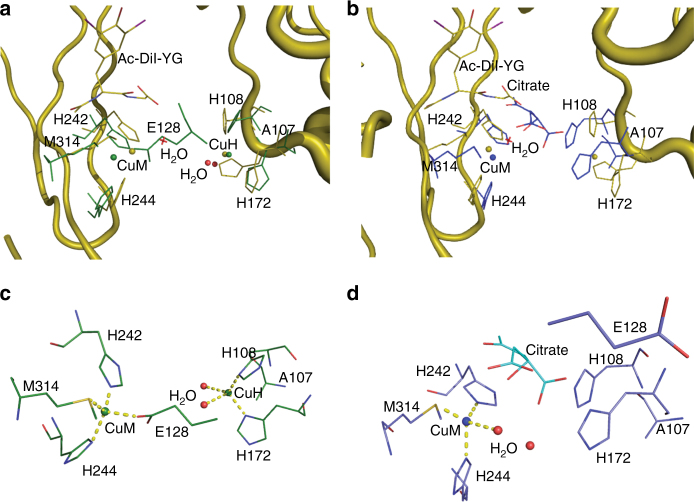
Cu coordination in the H107A-PHM mutant structure crystallized in the presence of citrate. There are two molecules A and D in the asymmetric unit (see text). **a** Superposition of wild-type PHM + peptide structure (PDB ID: 1OPM) shown in olive with H107A-PHM-cit-molecule A (green). Peptide-Acetyl-di-iodotyrosyl glycine (Ac-DiI-YG) in wild-type PHM is shown in olive. Cu_H_ and Cu_M_ are present in molecule A and are shown as green spheres. **b** Superposition of wild-type PHM + peptide with H107A-PHM-cit-molecule D (blue). Cu_H_ is absent in molecule D and CuM is shown as blue sphere. **c** Expanded view showing Cu coordination in H107A-PHM-cit-molecule A. Cu_H_ shows a tetrahedral geometry and Cu_M_ is coordinated by E128 in place of H_2_O. **d** Expanded view showing Cu coordination in H107A-PHM-cit-molecule D. Coordination of Cu_M_ in molecule D is similar to that of wild-type

Crystals of H107A-PHM (PDB ID: 5WJA) obtained from solutions containing 1–3 mM citrate as an additive diffracted to 2.3 Å resolution and contained two molecules in the asymmetric unit (Fig. 2, Table 1). These two molecules (Fig. 2a, b, molecules A and D, respectively) have significantly different conformations. In molecule A, both copper sites contain Cu as in wild-type PHM (Fig. 2a). However, even though no citrate is bound, a conformational change of the loop Cys126–Thr130 brings Glu128 in close proximity to the Cu_M_ site and unlike in wild-type PHM, the copper in the Cu_M_ site in this molecule is coordinated by Met314, His242, His244, and E128 takes the place of H_2_O as the fourth ligand (Fig. 2c). Furthermore, in this monomer, the Cu^2+^ present at the Cu_H_ site has a coordination different from that of the wild-type PHM. (The possibility of this being another metal can be ruled out based on the preparation of the crystals and the results of previous experiments: neither Ca, Mg, Zn, Cd, Co, Fe, Mn, Ni, Mo, Ba, V, Se, nor Si can replace Cu^41–45^.) The geometry is tetrahedral and is composed of the remaining two histidines (H108 and H172) plus two water molecules (Fig. 2c). In contrast, in molecule D, no density is found for Cu_H_ (Fig. 2b). Instead, the two histidine residues from the former Cu_H_ site interact with a bound citrate which, in turn, has a weak interaction with Cu_M_ mediated by H_2_O (Fig. 2d). Citrate binds close to the empty site of Cu_H_ and interacts directly with H108 and H172; the carboxylate of the citrate partially occupies the space that in wild-type PHM forms the peptide binding site (Fig. 2d). In addition, there is a movement of the loop spanning residues 45–53.

### Structure of the H108A-PHM mutant

**Fig. 3:**
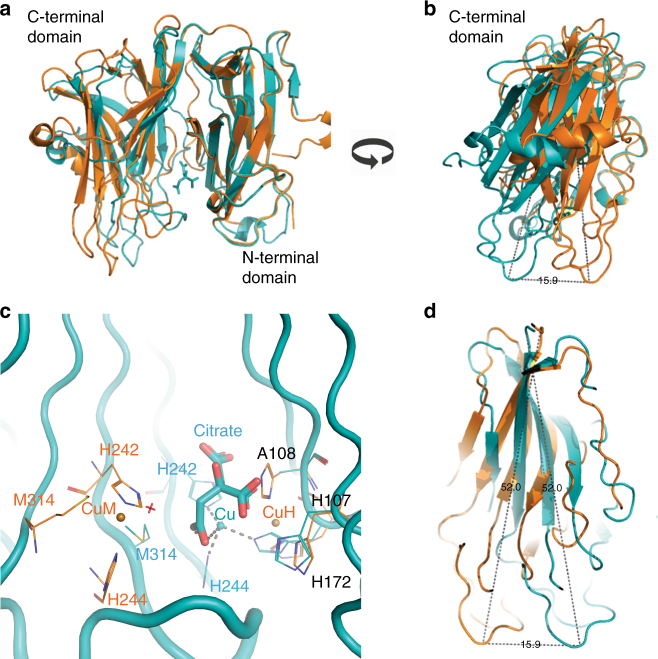
Cu coordination in the structure of the H108A-PHM mutant crystallized in complex with citrate. **a** Superposition of wild-type PHM structure (PDB ID: 1PHM; shown in orange) with the H108A-PHM-cit structure (PDB ID: 6ALA; teal) showing a rotation of the C-subdomain (aa 196–357) with respect to the N-subdomain (aa 53–195). The ligands of the Cu_H_ and Cu_M_ are combined to form a single Cu site. **b** A rotated 90° around the vertical axis. The distance between the T301 Cα of each structure shows a displacement of 15.9 Å. **c** Close-up of the Cu site in the H108A-PHM-cit (teal) showing a tetrahedral coordination with H242, H107, H244, and an oxygen of the citrate as ligands. **d** Close up of the overlap of residues 194–357 of wild-type PHM with those of H108A-PHM-cit. The C-subdomain of H108A-PHM-cit displays a rotation of about 18° toward the N-subdomain compared to the arrangement in wild-type PHM

PHM H108A (PDB ID: 6AO6) crystallizes with the same unit cell and space group as wild-type PHM. In the structure, determined to 3.0 Å resolution, H108A-PHM shows no major conformational changes compared to wild-type PHM (Supplementary Fig. 1C) even though there is no electron density for copper at the Cu_H_ site (Fig. 1d, Table 1). Crystals of H108A-PHM (PDB ID: 6ALA) obtained in a buffer containing citrate (3 mM) diffracted to 2.5 Å resolution and have a different space group and cell dimensions from those of wild-type PHM and H107A-PHM-cit (Fig. 3, Table 2). In these crystals there are two identical molecules with a bound citrate in the asymmetric unit present in a conformation significantly different from that of wild-type PHM. When the N-subdomain of the H108A-PHM-cit structure is aligned with that of wild-type PHM, the C-subdomain displays a rotation of ~18° toward the N-subdomain, compared to its position in the wild-type PHM structure. As a consequence of this hinge movement, several secondary elements of the C-subdomain move as much as 15 Å closer to the N-subdomain (Fig. 3b). These changes result in a complete reorganization of the coordination of the copper in Cu_M_. The two histidines, His 242 and His 244, remain coordinated to the copper, but Met 314 and the fourth ligand in wild-type PHM (a water molecule) do not bind to the ion. Of the two vacated Cu^2+^ coordination positions, one is occupied by the 2-carboxylate of citrate, and the other, surprisingly, by the N_ε_ of His 107, originally a copper ligand of the Cu_H_ site (Fig. 3c). In the wild-type PHM structure, the distance between His242 N_ε_ and His107 N_ε_ is 11.7 Å, while it is 3.9 Å in the H108A-PHM-cit structure (Fig. 3). The participation of His 107 in the coordination of Cu^2+^ at the Cu_M_ site brings the two domains of PHM closer together by approximately 7.8 Å.

**Table 2:** Data collection and refinement statistics

	Apo-PHM+Peptide PDB ID: 5WM0	H108A-PHM+Peptide PDB ID: 6AY0	H172A-PHM+Peptide PDB ID: 6AN3	H108A-PHM+Citrate PDB ID: 6ALA
*Data collection*				
Space group	P2_1_2_1_2_1_	P2_1_2_1_2_1_	P2_1_2_1_2_1_	C121
Cell dimensions				
* a*, *b*, *c* (Å)	59.09, 65.93, 69.80	58.80, 66.53, 70.21	59.02, 66.84, 70.24	171.37, 52.46, 116.45
* α β γ* (°)	90, 90, 90	90, 90, 90	90, 90, 90	90, 128.74, 90
Resolution (Å)	50–2.4 (2.44–2.40)	50–2.50 (2.54–2.50)	48.42–2.05 (2.10–2.05)	29.05–2.57 (2.65–2.59)
*R*_sym_	0.075 (0.329)	0.063 (0.129)	0.051(0.145)	0.116 (0.037)
*I* / σ*I*	35.75 (2.77)	48.10 (13.64)	45.98 (5.83)	8.8 (2.00)
CC(1/2)	NA	NA	NA	0.97 (0.65)
Completeness (%)	99.2(88.1)	94.5(69.0)	95.6(70.6)	96.8 (81.2)
Redundancy	5.5(2.6)	6.2(5.5)	5.4(2.0)	3.0 (2.8)
No. unique reflections	11068	9437	17975	25240
No. total reflections	60812	58408	93871	74724
Source	FR-E Superbright	FR-E Superbright	FR-E Superbright	APS Beamline 23-ID-B
Wavelength (Å)	1.54	1.54	1.54	1.033
*Refinement*				
Resolution (Å)	47.93–2.4 (2.46–2.00)	48.30–2.6 (2.66–2.60)	48.43–2.05 (2.10–2.05)	90.83–2.59 (2.65–2.59)
No. reflections	10468 (682)	8153 (554)	16297 (909)	23911 (1716)
*R*_work/_ *R*_free_	0.20/0.28 (0.25/0.27)	0.19/0.26 (0.20/0.27)	0.20/0.28 (0.20/0.27)	0.18/0.23 (0.26/0.34)
No. atoms				
Protein	2418	2418	2397	4737
Ligand/ion	—	1	7	26
Water	27	31	176	190
B-factors				
Protein	52.01	37.55	34.7	32
Ligand/ion	—	69.6	51.0	42.1
Water	41.2	29.12	42.6	28.6
R.m.s deviations				
Bond lengths (Å)	0.017	0.014	0.016	0.015
Bond angles (°)	1.93	1.81	1.87	1.89

Although citrate was added to the crystallization medium just to promote diffraction to higher resolution^46,47^, binding of citrate to the mutant PHMs could be related to the binding of fumarate to dopamine β-hydroxylase (DBH), a known DBH enhancer^48^. Four of the carbons of citrate, besides the absence of the double bond and the hydroxyl, are isosteric with those of fumarate.

### Structure of the H172A-PHM mutant

Crystals of the H172A-PHM mutant (PDB ID: 6AMP) have the same space group as wild-type PHM and highly similar cell dimensions and structure (RMSD 0.57 Å for 271 α-carbons). Even though the molecule does not contain copper in the Cu_H_ site (Fig. 1e), copper at the Cu_M_ site has the same coordination and geometry as the wild-type. The Cu_H_ site displays only the minimum changes compatible with the mutation and the absence of its copper (RMSD 0.61 Å) (Fig. 1e, Table 1). The N- and C-subdomains come closer by a small displacement (<0.5 Å) (Supplementary Fig. 1D).

### Binding of peptide substrate

**Fig. 4:**
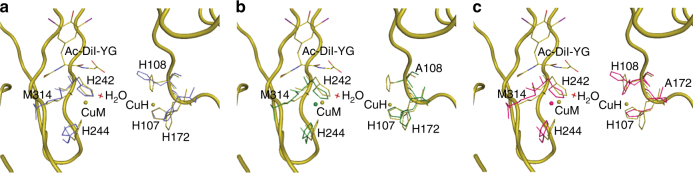
Binding of a peptide substrate to apo-PHM and to two PHM mutants-H108A and H172A. Superposition of wild-type PHM + peptide structure (olive) with apo-PHM in light blue (**a**), H108A-PHM in dark green (**b**), and H172A-PHM in pink (**c**). The peptide N-Acetyl-di-iodotyrosyl glycine (Ac-Di-I-YG) in wild-type PHM is shown in olive. No peptide bound to apo-PHM nor to either of the two mutants

The structure of oxidized wild-type PHM with the substrate N-acetyl-di-iodotyrosyl glycine (N-Ac-di-I-YG) bound has been determined previously^12^. To assess whether the copper ions are required for substrate binding, crystals of apo-PHM (PDB ID: 5WM0) were soaked in solutions containing 1 mg/mL of N-Ac-di-I-YG for up to 12 h. Data were collected from these crystals which diffracted to 2.4 Å resolution and the structure, refined to an *R*_factor_/*R*_free_ = 0.20/0.28, shows no density for the bound peptide (Fig. 4a, Table 2).

Similarly, the refined structures of crystals of the H108A (PDB ID: 6AY0) and H172A (PDB ID: 6AN3) mutants grown without citrate and soaked in solutions of mother liquor containing 1 mg/mL of N-Ac-di-I-YG for 2–12 h showed no density corresponding to the bound peptide (Fig. 4b, c, Table 2), suggesting that the presence of both copper ions is required for substrate binding. Co-crystallization with solutions containing 1 mg/mL peptide resulted in crystals that again did not show density for the bound peptide (Supplementary Fig. 2).

### Comparison of DBH structure with PHM

**Fig. 5:**
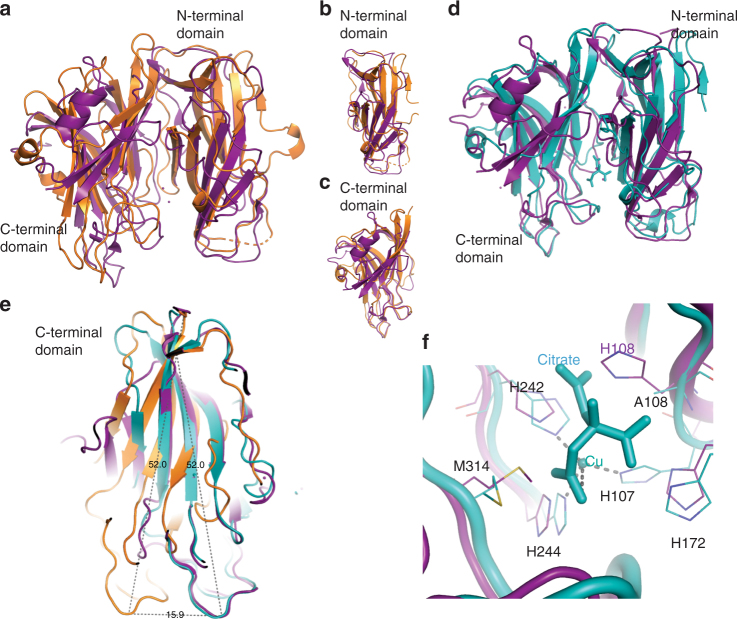
Structural comparison of dopamine β-hydroxylase (DBH) structure with wild-type PHM and H108A-PHM in complex with citrate. **a** Alignment of DBH (in purple) with wild-type PHM (in orange), N-terminal domains (**b**), and C-terminal domains (**c**). **d** Alignment of DBH with H108A-PHM in complex with citrate (in teal). **e** Hinge rotation of C-terminal domains toward N-terminal domains by ~18° in DBH and H108A-PHM in complex with citrate compared to wild-type PHM. **f** Alignment of the individual active site residues of chain A of hDBH with those of H108A-PHM in complex with citrate

DBH contains a catalytic domain (DBH-cat; residues 209 to 507) that is related in sequence (human DBH to rat PHM—29% identity and 44% similarity), copper content, structure^49^, and mechanism^50–52^ to PHM (Fig. 5a). Similar to PHM, DBH-cat has two subdomains—N-terminal (residues 209 to 356) and C-terminal (residues 357 to 507)—each containing a copper in the active form of the enzyme (also called Cu_H_ and Cu_M_). The reported crystal structure of DBH (PDB ID 4ZEL; 2.9 Å resolution) contains two molecules in the asymmetric unit, referred to as molecule A and molecule B. Only one of the four possible copper ions (Cu_M_ in molecule A) is reported in the PDB, albeit with low occupancy (reflected by the high B = 151.88). The structures of the individual DBH-cat subdomains are highly similar between the two molecules in the asymmetric unit (RMSD: N-terminal 0.73 Å for 141 Ca, C-terminal 0.21 Å for 150 Ca) even though the conformations of the DBH-cat domains in the two molecules are quite different (RMSD 4.10 Å for 260 Ca). In DBH molecule A, the two subdomains come closer together (closed arrangement), shortening significantly the distance between the putative Cu_H_ and the Cu_M_ sites (distance between DBH Cu_H_ and Cu_M_ sites is about 4–5 Å while in wild-type PHM, this distance is 11.7 Å). In DBH molecule B, the arrangement of the two subdomains is similar to that of wild-type PHM, making the distance between the putative Cu_H_–Cu_M_ sites ~11 Å (Supplementary Fig. 3).

The structures of the subdomains of DBH-cat are similar to the equivalent subdomains of wild-type PHM. The N-terminal subdomains of DBH molecules A and B have RMSDs of 1.33 Å (87 Ca) and 1.39 Å (92 Ca) with the N-terminal subdomain of PHM, respectively (Fig. 5b); the RMSDs for the C- terminal subdomains are 0.85 Å (85 Ca) and 0.86 Å (85 Ca) (Fig. 5c), respectively. Nevertheless, the overall conformation of both DBH-cat molecules in the crystal structure is different from that observed in the structure of wild-type PHM and the highly similar apo-PHM. Molecule A is the most different (RMSD 3.21 Å for 219 Ca). Although, the DBH-cat of molecule B is more similar to wild-type PHM, it still shows significant differences (RMSD 2.49 Å for 213 Ca).

The arrangement of subdomains in the DBH-cat of molecule A (closed) is highly similar to that of the H108A-PHM-cit structure (RMSD 1.19 Å for 195 Cα), including the position of the single copper ion of each structure (difference in position of the copper atom in the two aligned structures <1.6 Å). The similarity of these two structures, which contain a single copper, suggests that they may represent one-copper intermediates in the assembly of the two-copper competent PHM.

## Discussion

The structures of metalloenzyme active sites are exquisitely tailored to their catalytic function. PHM is an example of a copper enzyme that utilizes highly evolved reaction chemistry to catalyze a difficult hydroxylation believed to proceed via cupric-superoxo-mediated radical chemistry. However, this catalytic reactivity must proceed within the confines of equally sophisticated cellular transport mechanisms designed to ensure that the enzymes are metalated selectively and in response to cellular signals. In mammalian cells, the copper homeostatic machinery utilizes a pathway comprised of importers, chaperones, and energy-driven membrane pumps that eventually results in metalation of the catalytic metal centers via transporter-enzyme complexes^53^. Thus, the overall functioning of the system requires a fine balance between the requirements for selective metalation and the structural determinants of catalytic function. It has been suggested that ATP7A, a P-type ATPase, is a major required component of the system that metalates the PHM catalytic center^53^. It is likely that a component of this copper transfer mechanism may involve shared ligand complexes at the H-center where one or more of its histidine copper-ligands do not coordinate the copper. To address these issues, it is necessary to determine (a) the structural elements at the active site that facilitate the catalytic chemistry and (b) the conformational landscape which enables the enzyme to mature from its apo to the fully metalated catalytically competent form.

Since the Cu_H_ site mutants exhibit dramatically reduced activity, their structures may provide clues to both of these objectives. As a step toward this goal, we have determined the structures of the apo-enzyme and three His variants at the H-center-H107A, H108A, and H172A, all without the addition to Cu^2+^ in the crystallization media. All the mutants have previously been characterized in solution for kinetic parameters, metal content, and structural integrity using steady-state kinetics, inductively coupled plasma–optical emission spectrometry (ICP–OES), emission paramagnetic resonance (EPR), and XAS^19,33,54–56^. All of the mutants appeared to bind substrate in their di-copper forms with *K*_m_ values between 3 and 18 μM (wild-type *K*_m_ which is 8.3 μM) using dansyl-tyr-val-gly as substrate. The decrease in activity was thus almost entirely due to the large (>150 fold) decrease in *k*_cat_ for this substrate^56^. In the structures with one or both coppers missing, no substrate binding was observed, either by soaking or co-crystallization.

It is clear that in solution, H-site mutants can exist in forms that retain copper and substrate binding capacity, and the finding that crystal forms exist for H108A and H172A that are isostructural with wild-type broadly confirms these findings. However, the crystallographic determination of other conformers that either lack copper at the H-site, or that exhibit different conformations and/or binding modes, suggests that the mutations stabilize alternative conformers that may be intermediates in catalysis or metal transfer chemistry. The crystal forms that lack copper at the H-site may be stabilized by crystal-packing constraints or have lost copper to the copper-free crystallization media because of the weaker affinity of Cu(II) for the histidine-depleted site. Notwithstanding the fact that this result was unexpected, and recognizing that the mutants in solution may retain their di-copper structures, the crystal structures reported herein provide an unprecedented window into the role of metal occupancy in modulating the conformational landscape of PHM.

While loss of both coppers in the structure of apo PHM has no significant effect on the conformation of the molecule, mutations that cause loss of copper from a single site appear to have significant effects. We have previously shown that PHM mutations at the Cu_M_ (M314I) site result in conformational changes and a reduction in thermal stability^14^. The modifications of the Cu_H_ site reported in the present paper also result in significant structural effects. In wild-type PHM, this site has an unusual geometry: Cu^2+^ is only coordinated by three histidines (His 107, His 108 and His 172) with a T-shaped geometry. The fourth coordination position has been shown to remain unoccupied even in the presence of high concentrations of strong copper liganding small molecules (nitrite, azide, CO)^15^, which is unusual, given the strong preference of cupric centers to adopt square or tetragonal geometry. Here, insights into the Cu_H_ site were gained by studying the effects of substituting each of the three histidine ligands by alanine, one at a time.

The structure of the H107A mutant crystallized in the same conditions as the wild-type is highly similar to that of wild-type PHM. The coordination of the copper at the Cu_M_ site remains unchanged and even the two remaining histidines at Cu_H_ site show only minor changes (Fig. 1c). Inclusion of 1–3 mM citrate, which produced crystals (H107A-PHM-cit) that diffracted to higher resolution, had unexpected effects on the structure of H107A-PHM and provided information about alternative conformations that can be adopted by this mutant (Fig. 2). H107A-PHM-cit has two molecules in the asymmetric unit. Interestingly, the copper sites of these two molecules (A and D) present in the same unit cell have very different configurations (Fig. 2), probably because citrate is only present in one of the molecules (molecule D). In molecule A, copper binds at the Cu_H_ site despite the absence of His 107 by retaining the coordination with His 108 and His 172 and adding two water molecules as ligands. Even though molecule A does not have a bound citrate, it shows a change in the conformation of the loop containing residues 126 to 130 that brings Glu 128 into the proximity of the Cu_M_ site (Fig. 2a). Molecule D, in contrast, has no copper at the Cu_H_ site and contains a bound citrate that bridges the two remaining histidines of the copperless Cu_H_ site to the Cu_M_ site (Fig. 2b). It is clear that these changes are a consequence of the combination of the two modifications: the substitution of His 107 and the presence of citrate. Loss of coordination by H107 allows the Cu_H_ site to adopt a tetrahedral coordination by the inclusion of two water molecules, implying that square planar geometry at the H site is destabilized relative to the tetrahedral alternative. This observation strengthens the case for a functional requirement for a non-reactive open coordination site in Cu_H_ that allows electron transfer but prevents binding of small molecules that could modify the electrochemical potential of the copper. How the particular coordination of wild-type PHM accomplishes this feat remains unexplained.

Crystals of H108A-PHM, despite crystallizing in the same space group as wild-type PHM with similar cell dimensions, diffract only to 3.0 Å resolution and the structure shows no density for the copper in the Cu_H_ site. In every other respect, the structure of this mutant is highly similar to that of the wild-type (RMSD 0.57 Å for 1220 atoms of the main chain). The situation is different for the crystals obtained in the presence of 1–3 mM citrate: they diffract to 2.5 Å, the space group and cell dimensions are different from those of the wild-type and contain two identical molecules in the asymmetric unit with bound citrate. Neither molecule contains copper in the Cu_H_ site. Compared to wild-type PHM, the C-subdomain shows an ~18° rotation around an axis that goes through residue 201. The 2-carboxylate of the bound citrate coordinates the copper at the Cu_M_ and leads to a rearrangement of the copper coordination sphere (Fig. 3). The two histidines, His 242 and His 244, remain liganded to the copper but Met 314 does not coordinate the copper any longer. The water that occupies the fourth ligand position in wild-type PHM is replaced, surprisingly, by the N_ε_ of His 107—which in wild-type PHM coordinates the copper at Cu_H_. This interaction requires that the N- and the C-domains come closer together. The changes in conformation that result in this approach are extensive. The most salient feature is the flattening of the β-sheet of the N-terminal domain closest to the central cavity, which involves straightening of strand 5, the strand that starts with residue 107. It is difficult to quantitate these changes but one possible measure of the effect of this straightening may be the change in the distance between the α-carbons of residues Met 314 and His 107. In wild-type PHM, the distance is 19.6 Å and it is reduced to 12.5 Å in the H108A-PHM citrate complex. A measure of the effects that these changes in coordination have in other sections of the β-sheet involved in the Cu_H_ site—i.e., strand 9—is reflected in the distance between Met 314 and His 172; this distance is 17.9 Å in wild-type PHM and 13.8 Å in H108A-PHM-cit. Other notable changes are those in the loop spanning residues 129 to 136. This loop connects strands 5 and 7, and although its local conformation remains unchanged from that in wild-type PHM, its overall position is shifted away from the C-terminal domain. Again, these changes seem to be due as much to the mutation as to binding of citrate. In any case, they reflect the range of possible conformations that the molecule can adopt.

The H172A-PHM mutant is highly similar in structure to the oxidized wild-type PHM (PDB ID: 1YIP). More surprising, although it has no copper in the Cu_H_ site, the conformation of the two remaining histidines in this site, His 107 and His 108, is almost unmodified from that of wild-type PHM and the Cu_M_ site retains the same coordination of the copper observed in wild-type PHM.

**Fig. 6:**
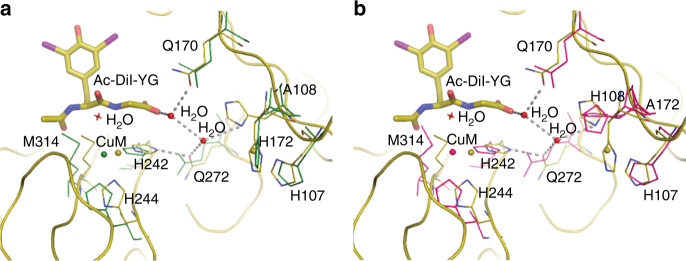
Interactions between Q170, Q272, H108, and two H_2_O molecules required to bind peptide substrate. Superposition of wild-type PHM + peptide (olive) with H108-PHM in dark green (**a**) and H172A-PHM in pink (**b**). A network of water molecules possibly required to bind peptide substrate is missing in the two mutant structures—H108A and H172A

To determine whether copper at both sites is required to bind peptide substrate, crystals of apo-PHM were both soaked and co-crystallized in mother liquor containing N-Ac-di-I-YG. This peptide binds to wild-type holo-PHM in both the oxidized and the reduced forms^41^. In contrast, the structure of apo-PHM determined with data collected from the N-Ac-di-I-YG soaked or co-crystallized crystals showed no additional density corresponding to the bound peptide, indicating that copper at both sites is required not only for the later steps of the catalysis but also for binding substrate. Furthermore, two water molecules that are a part of a network formed by Q170, Q272, H108, and the peptide substrate are present in wild-type PHM + peptide structure (PDB ID: 1OPM) but absent in H108A-PHM and H172A-PHM (Fig. 6a, b). These water molecules are possibly required for the peptide to bind to the enzyme. In the H108A and H172A mutants, lack of copper in the Cu_H_ site could prevent the two water molecules from forming the network required for peptide binding. In the wild-type enzyme, substrate binding has been shown to induce a new mode of CO binding at the M-center which lowers the C≡O infrared stretching frequency and suggests electronic activation of the diatomic ligand. This process does not occur in the mutants, consistent with a lack of substrate binding although clearly other factors could also be responsible^57^.

The recently published 2.9 Å resolution structure of apo human dopamine β–hydroxylase (hDBH, PDB ID: 4ZEL)^49^ has two monomers in the asymmetric unit (molecules A and B) with different conformations. In chain A, the two subdomains display a closed conformation in which the C-subdomain is closer to N-subdomain (hinge rotation of ~18°) resulting in a structure very similar to that observed in our H108A-PHM structure in complex with citrate (Fig. 5d, e). Furthermore, the individual active site residues of chain A of hDBH align closely with those of H108A-PHM in complex with citrate (Fig. 5f). The single copper ions present in both structures have similar coordination and are in approximately the same positions in the aligned structures (distance ≤1.6 Å). The other chain of the hDBH crystals (molecule B), however, shows an open active site similar to that of wild-type PHM (Fig. 5a). Christensen and co-workers^49^ modeled a copper in the Cu_M_ site of chain A and used the positions of the Cu-liganding residues in chains A and B to infer the position of the three other coppers. Based on this, the authors suggested the possibility that Cu_H_ and Cu_M_ could come as close as 4–5 Å to each other during the catalytic cycle, close enough to form a binuclear copper center^49^. However, the fact that this conformation was only seen in structures with a single copper ion (H108A-PHM-cit and hDBH chain A) is more compatible with the conclusion that having two bound coppers has an important structural role: locking PHM (and probably DBH) in the catalytically competent conformation. This role is unusual for a catalytic redox active metal^58^. The closed structures observed in hDBH and H108A-PHM-cit may represent intermediates in the loading of copper ions during the final assembly. This is an attractive hypothesis since the molecule with only one copper could have access to a number of alternative conformations which may assist in loading the second copper, and/or may be required for recognition of its cognate transfer partner^32,33,59^.

All PHM structures determined previously (reduced, oxidized, with and without ligands, and in a precatalytic complex) have had copper ions in both sites including the Cu_M_ mutant M314I^14^. In all these structures, the distance between the two coppers is approximately ~11 Å^12,14,15,41^. This observation, together with the fact that PHM crystals can carry out sustained catalysis, supports a mechanism that does not require a large conformational change to bring the copper ions into close enough proximity for a direct electron transfer between the ions.

The structures of the Cu_H_ site mutants (H107A, H108A, and H172A), even those with unoccupied copper sites, are all found in the open conformation. The incorporation of citrate in the crystallization media as an additive had a positive effect on the resolution but, surprisingly, resulted in structures that show significant changes with respect to wild-type, providing a glimpse into the changed conformational landscape that results from the absence of one copper.

## Methods

### Protein expression, mutagenesis, and purification

Stable cell lines secreting PHM and its mutants, H107A, H108A, and H172A, were created using our established methods^12,13,33,37,60^. Briefly, CHO cell lines were transfected with a vector encoding the protein of interest and *dhfr*; the cells were selected by growth in αMEM plus 10% dialyzed fetal bovine serum, subcloned and tested for enzyme expression and clonality until stable, clonal lines were obtained. Typically, it took several months to obtain a cell line in which at least 1–2% of total protein synthesis was devoted to the protein of interest, and in which the cell doubling time remained less than a day^37,60^. Wild-type and mutant cell-lines were thawed from freezer stock into T75 flasks with 20 mL of DMEM/F12 medium containing 10% FCII serum (Fisher). At 80% confluence, the cells were passed into five NUNC triple flasks (500 cm^2^ area per flask) which were then grown until the cells were 80% confluent. Cells were trypsinized and resuspended in 50 mL medium containing 10% FCII serum and then inoculated into the extra-capillary space (ECS) of a Hollow Fiber Bioreactor (Fibercell Systems 4300-C2008, MWCO 5 kD, 3000 cm^2^ surface area) precultured with 2 L of 50 mM PBS pH 7.35 and 2 L of DMEM/F12 10% FCII serum^61–63^. Individual bioreactors containing each of the mutants were fed with DMEM/F12/10% FCII serum for 2–3 weeks, after which the serum level was lowered to 0.5% FCII serum. Thereafter, the bioreactors were fed with 0.5% serum-containing medium every other day and spent medium (20 mL) from the ECS was harvested and frozen at −20 °C for subsequent purification^63^.

### Preparation of samples for apo and mutant proteins

Purified enzymes (wild-type and mutants) were dialyzed against 20 mM sodium phosphate buffer, pH 8.0. The apo-protein isolated from recombinant CHO cells contained no copper, and was used without further addition of copper. Mutant proteins, H107A, H108A, and H172A-PHM, were also devoid of copper as isolated and were subsequently reconstituted with cupric sulfate by slow addition of 2.5 molar equivalents Cu(II) per protein followed by two cycles of dialysis to remove unbound cupric ions. Protein Concentrations were determined using OD280(1%) = 0.980 on a Cary 50 spectrophotometer. Copper concentrations were determined using a Perkin-Elmer Optima 2000 DV inductively coupled plasma optical emission spectrometer.

### Crystallization

Crystals of apo-PHM and PHM mutants, H107A, H108A, and H172A, were prepared using hanging drop vapor diffusion. Conditions for crystallization were similar to those used for wild-type PHM^12^. In brief, drops were made by mixing 1 µL of PHM (14–17 mg/mL) with 1 µL of 0.1 M Tris HCl pH 8.5, 0.54 M MgCl_2_, and 19–24% PEG 4000 by vapor diffusion. Prior to data collection, apo-PHM, H108A, and H172A mutant crystals were soaked with 1 mg/mL N-Acetyl-di-iodo phenylalanyl glycine or N-Ac-di-I-tyrosyl glycine peptide for 2 h. Crystals of H107A-PHM and H108A-PHM in complex with citrate were grown from the condition described above using 1–3 mM citrate.

### Data collection, structure determination, and refinement

Data for apo-PHM, H108A-PHM, and H172A-PHM crystals were collected on an FR-E Super-Bright Rigaku (Americas Corporation, The Woodlands, TX) copper rotating anode x-ray generator as the source with a DECTRIS Pilatus 3R 200K-A detector at 100 K. Data for H107A-PHM were collected at beam line 17-ID-1, NSLSII, BNL on a DECTRIS Eiger 6M detector. Data for H107A-PHM in complex with citrate were collected on beam line 23-ID APS. Data were indexed, integrated, and scaled with HKL3000, XDS, and Fast DP. H172A-PHM structure was determined by molecular replacement using wild-type PHM (PDB ID: 1PHM) as a template. All the other structures were determined by Fourier synthesis using the initial structure. Each of the models was rebuilt and refined using alternate cycles of Coot and restrained refinement with Refmac5 in the CCP4 suite^64–66^. Final models were validated using Coot^64^ and Molprobity^67^. Figures for the structures were prepared with PyMOL^68,69^. RMSD between models was calculated using the LSQ function in Coot.

### Data availability

Atomic coordinates and structure factors are available at the Protein Data Bank with the following PDB IDs for the corresponding proteins: apo-PHM (PDB ID: 5WKW), H107A-PHM (PDB ID: 6ALV), H108A-PHM (PDB ID: 6AO6), H172A-PHM (PDB ID: 6AMP), H107A-PHM in complex with citrate (PDB ID: 5WJA), H108A-PHM in complex with citrate (PDB ID: 6ALA), apo-PHM in complex with peptide (no peptide observed, PDB ID: 5WM0), H108A in complex with peptide (no peptide observed, PDB ID: 6AY0), and H172A in complex with peptide (no peptide observed, PDB ID: 6AN3) were deposited in the Protein Databank. All other data that support the findings of this study are available from the corresponding authors upon reasonable request.

## Electronic supplementary material

Supplementary Information

## References

[1] Bradbury AF, Smyth DG (1988). Peptide amidation: evidence for multiple molecular forms of the amidating enzyme. Biochem. Biophys. Res. Commun..

[2] Bradbury AF, Smyth DG (1991). Peptide amidation. Trends Biochem. Sci..

[3] Eipper BA, Milgram SL, Husten EJ, Yun HY, Mains RW (1993). Peptidylglycine a-amidating monooxygenase: a multifunctional protein with catalytic, processing and routing domains. Protein Sci..

[4] Merkler DJ (1994). C-terminal amidated peptides: production by the in vitro enzymatic amidation of glycine-extended peptides and the importance of the amide to bioactivity. Enzyme Microb. Technol..

[5] Prigge ST, Mains RE, Eipper BA, Amzel LM (2000). New insights into copper monooxygenases and peptide amidation: structure, mechanism and function. Cell. Mol. Life Sci..

[6] Kumar D (2016). Early eukaryotic origins for cilia-associated bioactive peptide-amidating activity. J. Cell. Sci..

[7] Attenborough RM, Hayward DC, Kitahara MV, Miller DJ, Ball EE (2012). A “neural” enzyme in nonbilaterian animals and algae: preneural origins for peptidylglycine alpha-amidating monooxygenase. Mol. Biol. Evol..

[8] Eipper BA (1991). Peptidyl-alpha-hydroxyglycine alpha-amidating lyase - purification, characterization, and expression. J. Biol. Chem..

[9] Kato I, Yonekura H, Okamoto H (1991). [Two enzymes concerned in peptide hormone alpha-amidation are synthesized from a single mRNA]. Seikagaku J. Jpn. Biochem. Soc..

[10] Stoffers DA, Green CB, Eipper BA (1989). Alternative mRNA splicing generates multiple forms of peptidyl-glycine alpha-amidating monooxygenase in rat atrium. Proc. Natl Acad. Sci. USA.

[11] Rudzka K (2013). Coordination of peroxide to the Cu(M) center of peptidylglycine alpha-hydroxylating monooxygenase (PHM): structural and computational study. J. Biol. Inorg. Chem..

[12] Prigge ST, Kolhekar AS, Eipper BA, Mains RE, Amzel LM (1997). Amidation of bioactive peptides: the structure of peptidylglycine alpha-hydroxylating monooxygenase. Science.

[13] Prigge ST, Kolhekar AS, Eipper BA, Mains RE, Amzel LM (1999). Substrate-mediated electron transfer in peptidylglycine alpha-hydroxylating monooxygenase. Nat. Struct. Biol..

[14] Siebert X (2005). The catalytic copper of peptidylglycine alpha-hydroxylating monooxygenase also plays a critical structural role. Biophys. J..

[15] Chufan EE (2010). Differential reactivity between two copper sites in peptidylglycine alpha-hydroxylating monooxygenase. J. Am. Chem. Soc..

[16] Klinman JP (2006). The copper-enzyme family of dopamine beta-monooxygenase and peptidylglycine alpha-hydroxylating monooxygenase: resolving the chemical pathway for substrate hydroxylation. J. Biol. Chem..

[17] Hess CR, Klinman JP, Blackburn NJ (2010). The copper centers of tyramine beta-monooxygenase and its catalytic-site methionine variants: an X-ray absorption study. J. Biol. Inorg. Chem..

[18] Evans JP, Ahn K, Klinman JP (2003). Evidence that dioxygen and substrate activation are tightly coupled in dopamine beta-monooxygenase. Implications for the reactive oxygen species. J. Biol. Chem..

[19] Evans JP, Blackburn NJ, Klinman JP (2006). The catalytic role of the copper ligand H172 of peptidylglycine alpha-hydroxylating monooxygenase: a kinetic study of the H172A mutant. Biochemistry.

[20] Chauhan S, Hosseinzadeh P, Lu Y, Blackburn NJ (2016). Stopped-flow studies of the reduction of the copper centers suggest a bifurcated electron transfer pathway in peptidylglycine monooxygenase. Biochemistry.

[21] Mueller GP, Driscoll WJ, Eipper BA (1999). In vivo inhibition of peptidylglycine-alpha-hydroxylating monooxygenase by 4-phenyl-3-butenoic acid. J. Pharmacol. Exp. Ther..

[22] Driscoll WJ (2000). Peptidylglycine-alpha-hydroxylating monooxygenase generates two hydroxylated products from its mechanism-based suicide substrate, 4-phenyl-3-butenoic acid. Biochemistry.

[23] Merkler DJ (2008). Substituted hippurates and hippurate analogs as substrates and inhibitors of peptidylglycine alpha-hydroxylating monooxygenase (PHM). Bioorg. Med. Chem..

[24] Langella E (2010). Probing the peptidylglycine alpha-hydroxylating monooxygenase active site with novel 4-phenyl-3-butenoic acid based inhibitors. ChemMedChem.

[25] Hess CR (2008). Hydroxylase activity of Met471Cys tyramine beta-monooxygenase. J. Am. Chem. Soc..

[26] Blackburn NJ, Hasnain SS, Pettingill TM, Strange RW (1991). Copper K-extended x-ray absorption fine structure studies of oxidized and reduced dopamine beta-hydroxylase. Confirmation of a sulfur ligand to copper(I) in the reduced enzyme. J. Biol. Chem..

[27] Pettingill TM, Strange RW, Blackburn NJ (1991). Carbonmonoxy dopamine beta-hydroxylase. Structural characterization by Fourier transform infrared, fluorescence, and x-ray absorption spectroscopy. J. Biol. Chem..

[28] Boswell JS, Reedy BJ, Kulathila R, Merkler D, Blackburn NJ (1996). Structural investigations on the coordination environment of the active-site copper centers of recombinant bifunctional peptidylglycine alpha-amidating enzyme. Biochemistry.

[29] Chen P, Bell J, Eipper BA, Solomon EI (2004). Oxygen activation by the noncoupled binuclear copper site in peptidylglycine alpha-hydroxylating monooxygenase. Spectroscopic definition of the resting sites and the putative CuIIM-OOH intermediate. Biochemistry.

[30] Rhames FC, Murthy NN, Karlin KD, Blackburn NJ (2001). Isocyanide binding to the copper(I) centers of the catalytic core of peptidylglycine monooxygenase (PHMcc). J. Biol. Inorg. Chem..

[31] Sarangi R (2006). X-ray absorption edge spectroscopy and computational studies on LCuO2 species: superoxide-Cu(II) versus peroxide-Cu(III) bonding. J. Am. Chem. Soc..

[32] Otoikhian A (2012). Lumenal loop M672-P707 of the Menkes protein (ATP7A) transfers copper to peptidylglycine monooxygenase. J. Am. Chem. Soc..

[33] Kline CD, Mayfield M, Blackburn NJ (2013). HHM motif at the CuH-site of peptidylglycine monooxygenase is a pH-dependent conformational switch. Biochemistry.

[34] Chen P, Solomon EI (2004). O2 activation by binuclear Cu sites: noncoupled versus exchange coupled reaction mechanisms. Proc. Natl Acad. Sci. USA.

[35] Chen P, Solomon EI (2004). Oxygen activation by the noncoupled binuclear copper site in peptidylglycine alpha-hydroxylating monooxygenase. Reaction mechanism and role of the noncoupled nature of the active site. J. Am. Chem. Soc..

[36] Crespo A, Marti MA, Roitberg AE, Amzel LM, Estrin DA (2006). The catalytic mechanism of peptidylglycine alpha-hydroxylating monooxygenase investigated by computer simulation. J. Am. Chem. Soc..

[37] Eipper BA, Quon AS, Mains RE, Boswell JS, Blackburn NJ (1995). The catalytic core of peptidylglycine alpha-hydroxylating monooxygenase: investigation by site-directed mutagenesis, Cu X-ray absorption spectroscopy, and electron paramagnetic resonance. Biochemistry.

[38] Kulathila R (1994). Bifunctional peptidylglcine alpha-amidating enzyme requires two copper atoms for maximum activity. Arch. Biochem. Biophys..

[39] Kolhekar AS, Keutmann HT (1997). Peptidylglycine alpha-hydroxylating monooxygenase: active site residues, disulfide linkages, and a two-domain model of the catalytic core. Biochemistry.

[40] Yonekura H (1996). Identification of the five essential histidine residues for peptidylglycine monooxygenase. Biochem. Biophys. Res. Commun..

[41] Prigge ST, Eipper BA, Mains RE, Amzel LM (2004). Dioxygen binds end-on to mononuclear copper in a precatalytic enzyme complex. Science.

[42] Eipper BA, Mains RE, Glembotski CC (1983). Identification in pituitary tissue of a peptide alpha-amidation activity that acts on glycine-extended peptides and requires molecular oxygen, copper, and ascorbic acid. Proc. Natl Acad. Sci. USA.

[43] Glembotski CC, Eipper BA, Mains RE (1984). Characterization of a peptide alpha-amidation activity from rat anterior pituitary. J. Biol. Chem..

[44] Murthy AS, Keutmann HT, Eipper BA (1987). Further characterization of peptidylglycine alpha-amidating monooxygenase from bovine neurointermediate pituitary. Mol. Endocrinol..

[45] Barber-Zucker S, Shaanan B, Zarivach R (2017). Transition metal binding selectivity in proteins and its correlation with the phylogenomic classification of the cation diffusion facilitator protein family. Sci. Rep..

[46] Friedmann D, Messick T, Marmorstein R (2011). Crystallization of macromolecules. Curr. Protoc. Protein Sci..

[47] Benvenuti M, Mangani S (2007). Crystallization of soluble proteins in vapor diffusion for x-ray crystallography. Nat. Protoc..

[48] Wimalasena DS, Jayatillake SP, Haines DC, Wimalasena K (2002). Plausible molecular mechanism for activation by fumarate and electron transfer of the dopamine beta-mono-oxygenase reaction. Biochem. J..

[49] Vendelboe TV (2016). The crystal structure of human dopamine beta-hydroxylase at 2.9 A resolution. Sci. Adv..

[50] Francisco WA, Knapp MJ, Blackburn NJ, Klinman JP (2002). Hydrogen tunneling in peptidylglycine alpha-hydroxylating monooxygenase. J. Am. Chem. Soc..

[51] Francisco WA, Merkler DJ, Blackburn NJ, Klinman JP (1998). Kinetic mechanism and intrinsic isotope effects for the peptidylglycine alpha-amidating enzyme reaction. Biochemistry.

[52] Osborne RL, Zhu H, Iavarone AT, Blackburn NJ, Klinman JP (2013). Interdomain long-range electron transfer becomes rate-limiting in the Y216A variant of tyramine beta-monooxygenase. Biochemistry.

[53] El Meskini R, Culotta VC, Mains RE, Eipper BA (2003). Supplying copper to the cuproenzyme peptidylglycine alpha-amidating monooxygenase. J. Biol. Chem..

[54] Bauman AT, Broers BA, Kline CD, Blackburn NJ (2011). A copper-methionine interaction controls the pH-dependent activation of peptidylglycine monooxygenase. Biochemistry.

[55] Jaron S, Mains RE, Eipper BA, Blackburn NJ (2002). The catalytic role of the copper ligand H172 of peptidylglycine alpha-hydroxylating monooxygenase (PHM): a spectroscopic study of the H172A mutant. Biochemistry.

[56] Bauman AT, Yukl ET, Alkevich K, McCormack AL, Blackburn NJ (2006). The hydrogen peroxide reactivity of peptidylglycine monooxygenase supports a Cu(II)-superoxo catalytic intermediate. J. Biol. Chem..

[57] Kline CD, Blackburn NJ (2016). Substrate-induced carbon monoxide reactivity suggests multiple enzyme conformations at the catalytic copper m-center of peptidylglycine monooxygenase. Biochemistry.

[58] Berg JM (1987). Metal ions in proteins: structural and functional roles. Cold Spring Harb. Symp. Quant. Biol..

[59] Kline CD, Gambill BF, Mayfield M, Lutsenko S, Blackburn NJ (2016). pH-regulated metal-ligand switching in the HM loop of ATP7A: a new paradigm for metal transfer chemistry. Metallomics.

[60] Kolhekar AS, Mains RE, Eipper BA (1997). Peptidylglycine a-amidating monooxygenase: an ascorbate-requiring enzyme. Methods Enzymol..

[61] Blackburn NJ, Rhames FC, Ralle M, Jaron S (2000). Major changes in copper coordination accompany reduction of peptidylglycine monooxygenase: implications for electron transfer and the catalytic mechanism. J. Biol. Inorg. Chem..

[62] Jaron S, Blackburn NJ (1999). Does superoxide channel between the copper centers in peptidylglycine monooxygenase? A new mechanism based on carbon monoxide reactivity. Biochemistry.

[63] Bauman AT, Ralle M, Blackburn NJ (2007). Large scale production of the copper enzyme peptidylglycine monooxygenase using an automated bioreactor. Protein Expr. Purif..

[64] Emsley P, Lohkamp B, Scott WG, Cowtan K (2010). Features and development of Coot. Acta Crystallogr. D Biol. Crystallogr..

[65] Potterton E, Briggs P, Turkenburg M, Dodson E (2003). A graphical user interface to the CCP4 program suite. Acta Crystallogr. D Biol. Crystallogr..

[66] Winn MD, Ashton AW, Briggs PJ, Ballard CC, Patel P (2002). Ongoing developments in CCP4 for high-throughput structure determination. Acta Crystallogr. D Biol. Crystallogr..

[67] Chen VB (2010). MolProbity: all-atom structure validation for macromolecular crystallography. Acta Crystallogr. D Biol. Crystallogr..

[68] DeLano, W. L. The PyMOL Molecular Graphics System (2002).

[69] Schrodinger, L. L. C. The PyMOL Molecular Graphics System, V.1.8 (2015).

