# Supplementary material for: Effects of copper occupancy on the conformational landscape of peptidylglycine α-hydroxylating monooxygenase
Source: Commun Biol. 2018 Jun 25;1:74. doi: 10.1038/s42003-018-0082-y (PMC6123673; doi:10.1038/s42003-018-0082-y)
Supplement: Supplementary file 1 — Supplementary Information [file 42003_2018_82_MOESM1_ESM.pdf]

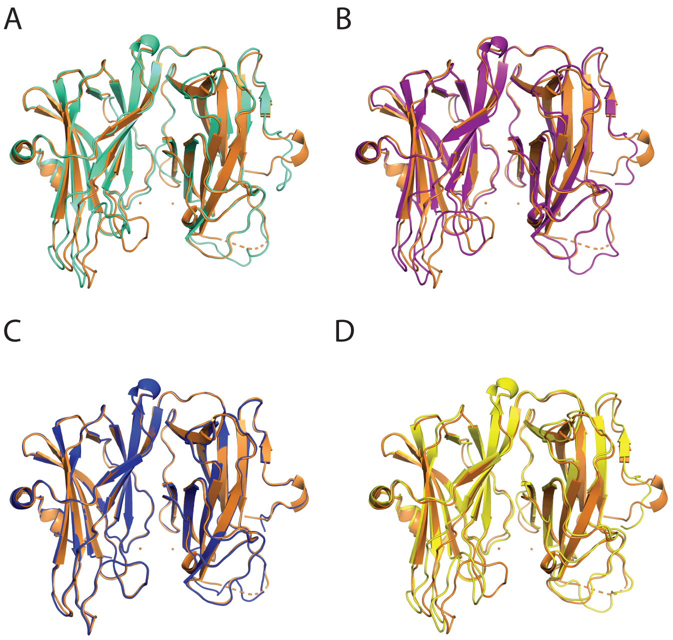

Supplementary Figure 1. Alignment of WT PHM (in orange) with apo-PHM (in cyan) (A), H107A-PHM (in magenta) (B), H108A-PHM (in blue) (C), H172A-PHM (in yellow) (D).

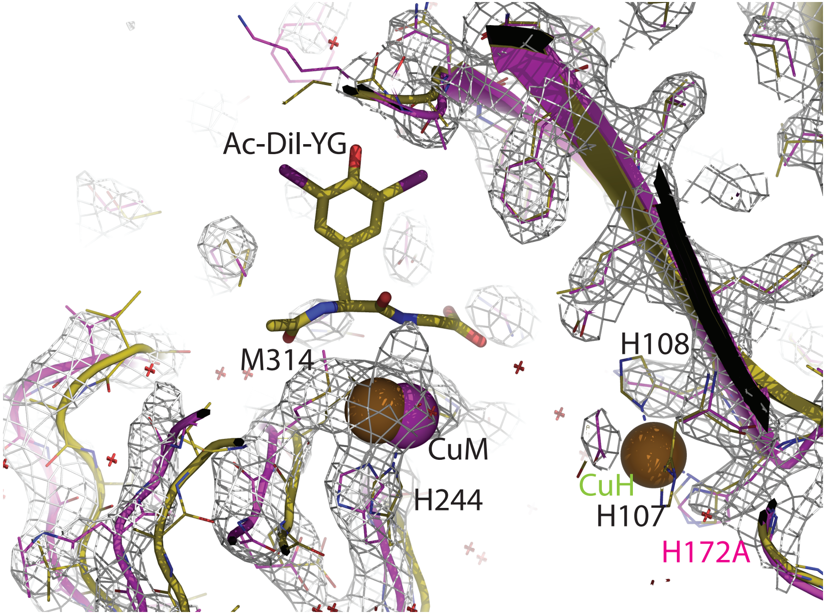

Supplementary Figure 2. Lack of electron density for the peptide in the structure of the H172A-PHM co-crystallized with peptide. H172-PHM is shown in magenta and WT-PHM in complex with Ac-DiI-YG shown in olive. Data for H172A-PHM in complex with peptide were collected at beam line 17-ID-1, NSLSII, BNL on an DECTRIS Eiger 6M detector.

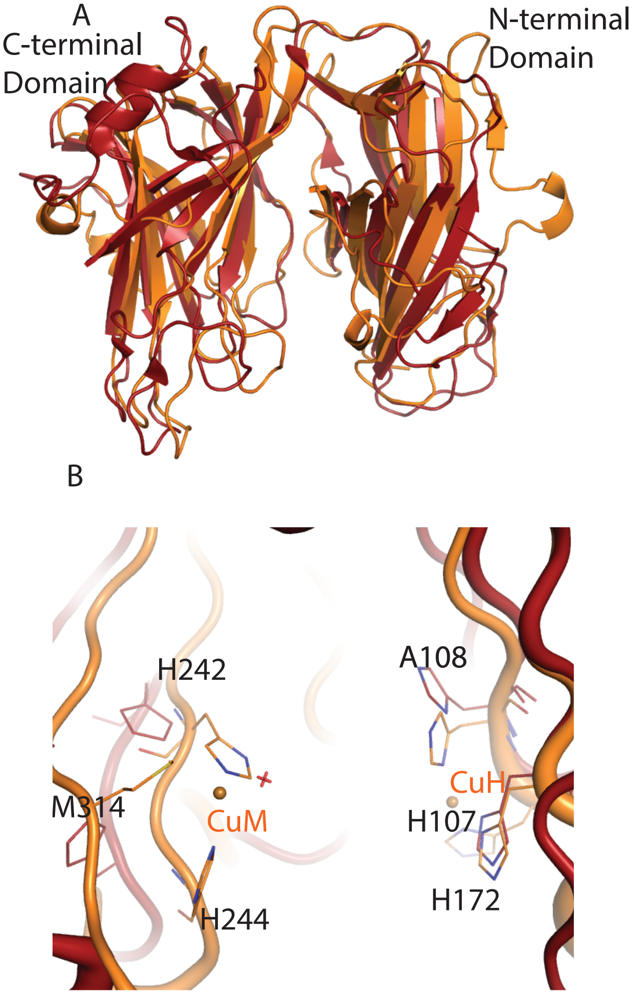

Supplementary Figure 3. A. Alignment of chain B of DBH (in dark red) with WT PHM (in orange), B. Alignment of individual active site residues of chain B of DBH (in purple) with WT PHM (in dark red).
